# Burden of Treatment in Children and Adolescents With Type 1 Diabetes Evaluated by Focus Groups

**DOI:** 10.1155/pedi/8833434

**Published:** 2025-06-12

**Authors:** Sophie Le Fur, Iva Gueorguieva, Kevin Perge, Fatiha Guémazi, Nathalie Frament, Natacha Bouhours-Nouet, Berthe Razafimahefa, Pascale Trioche-Eberschweiler, Ramona Nicolescu, Patricia Pigeon-Kherchiche, Fabienne Dalla Vale, Claire Rodet, Alice Bonin, Nadège Bourvis, Pierre Bougnères

**Affiliations:** ^1^GET-DOC Association, Paris, France; ^2^Pediatric Endocrine Unit, Hôpital Jeanne de Flandre, CHU Lille, Lille, France; ^3^Department of Pediatric Endocrinology, Hôpital Femme Mère Enfant, CHU Lyon, Bron, France; ^4^Center of Pediatric Diabetology, Mulhouse, France; ^5^Department of Pediatric Endocrinology and Diabetology, CHU of Angers, Angers, France; ^6^Department of Pediatrics, Center Hospitalier Intercommunal Toulon la Seyne (CHITS), Toulon, France; ^7^Department of Pediatrics, Hôpital Antoine-Béclère, CHU Paris Saclay, Clamart, France; ^8^Department of Pediatrics, Hôpital Nord, Saint-Etienne, France; ^9^Department of Pediatrics, CHU Réunion, Saint Denis de la Réunion, France; ^10^Institut Saint-Pierre, Palavas-les-Flots, France; ^11^Department of Diabetes Development, Elivie, Villeurbanne, France; ^12^Medical Department, Insulet, Paris, France; ^13^Maison des Adolescents du Var, Toulon, France; ^14^Department of Child and Adolescent Psychiatry, Center Hospitalier Intercommunal Toulon la Seyne (CHITS), Toulon, France; ^15^Centre des Princes, 31 rue des Princes, Boulogne-Billancourt, France

**Keywords:** adolescents, burden of treatment, children, focus groups, type 1 diabetes

## Abstract

**Objective:** Taking into account the burden of treatment (BOT) should favor psychological fulfillment and adherence of young patients to treatment, which largely determines the quality of type 1 diabetes (T1D) control. To identify BOT components, the Ariane study carried out a focus group survey among 84 children and adolescents with T1D aged 12.6 ± 3.7 years.

**Research Design and Methods:** Focus groups were organized in 10 pediatric diabetes centers, a qualitative research method that brings together a small group of patients to express their perception of treatment and answer questions in a moderated nonmedical setting.

**Results:** A total of 3640 verbatim voicing children's concerns were recorded, transcribed, and analyzed by five working groups composed of pediatric diabetologists, specialized nurses, adults with childhood-onset T1D, and two groups from the civil society. Each group studied the verbatim separately to extract 24 main concerns summarizing BOT. These concerns fell into two distinct categories: concerns about physical, material, and care organization (*N* = 15) or psychological concerns (*N* = 9). A BOT score summed the number of concerns of each patient. The mean BOT score was 7.4 ± 3.3 (range 1–18). Gender had a prominent influence on concerns (*p*=0.002).

**Conclusions:** The identification of common concerns expressed through focus groups provides a new tool for estimation of BOT in childhood T1D.

## 1. Introduction

Type 1 diabetes (T1D) induces by itself a burden inevitably linked to the diagnosis of an incurable disease, to which the burden of treatment (BOT) adds its own concerns. As defined by V. Montori, “the burden of treatment refers to the demands that healthcare professionals place on patients, and the implications this has on their quality of life” [1]. While many practitioners have applied this principle for a long time without naming it, the concept of minimally disruptive medicine (MDM) for patients with chronic illnesses has appeared in the medical literature only at the end of the 2000s [1]. MDM is an approach that minimizes healthcare disruption on patients' lives and the BOT. Measures of BOT and MDM have been developed notably in adults with diabetes[2, 3]. Although therapeutic progress and new technology ease some of the load and allow children with T1D to achieve tighter glycemic management, the burden of diabetes care, including digital management, remains significant[4, 5]. Indeed, BOT includes putting self-management recommendations into practice, taking multiple insulin doses through subcutaneous injections or catheters, wearing various devices, following dietary rules and planning meals, measuring multiple glucose levels every day, calculating insulin doses adjusted to the meals and the day's events, and enduring unavoidable hypoglycemic reactions. If they want to reduce the unavoidable physical and mental burden associated with T1D management, doctors need to know which aspects of treatment are the most burdensome for a given patient and take this individual information into account in their prescriptions and recommendations.

The BOT of several chronic diseases, including diabetes, has been widely studied in adults [6], much less in children (see Discussion). One of the reasons for this is methodological, as involving children in the strict conditions required for such a study is particularly difficult. Indeed, a reliable assessment of the BOT in children and adolescents with T1D requires an interviewing method that combines sincerity, freedom of expression, and stays focused on the perception of treatment.

Even if the primary goal of T1D treatment does remain short- and long-term metabolic efficacy, the quest for minimally disruptive care should gain importance in the therapeutic relationship with the patient, notably in such vulnerable age groups as childhood and adolescence. Taking into account the BOT in daily life is desirable for psychological fulfillment, as well as for personal adherence to treatment, which largely determines the quality of T1D control. This is particularly true at a time when intensive treatment is taking an ever-greater place in the daily routine, at home, at school, and on vacation. Physicians' and parents' choice of therapeutic devices and demanding procedures should consider the BOT of the young patients, whose opinion is not always solicited with sufficient attention.

Exploring the dynamics of BOT in pediatric ages is the aim of the Ariane study. For this purpose, we used focus groups, a qualitative research method that brings together a small group of patients to express their feelings and answer questions in a moderated nonmedical setting [7–15]. The questions were designed to shed light on treatment perception, our primary goal being to identify the components of the BOT in childhood T1D.

## 2. Materials and Methods

### 2.1. Ariane Group

The Ariane Group is composed of 10 experienced diabetologists-pediatricians, each leading a diabetes center, two clinical research associates (SLF and NF), a nurse specialized in home care for pediatric T1D (CR), a pharmacist from the insulin pump industry (AB), and a child psychiatrist (NB). A diagram of the Ariane survey is shown in Figure S1.

### 2.2. Patients

In 2023, 84 patients aged 5–20 years were interviewed in groups of 4 on average. Inclusion criteria were children over 5 years old and adolescents with T1D, selected by the Ariane pediatricians to be reasonably outspeaking and representing a variety of personalities, socio–economic status, and treatment methods. Participants' characteristics are described in Table 1. Five children aged 5–6 years were included as the youngest patients, after checking that they were able and willing to express themselves. The research protocol was approved by the Ethics Committee of Ile de France (DC-2008-693) and the Commission Nationale Informatique et Libertés (DR-2010-0035). The ClinicalTrial.gov identifier was NCT02212522. Patients provided written informed consent for participation in the study.

### 2.3. Focus Groups

Focus groups were held at 10 pediatric diabetes centers of the Ariane network. They were conducted according to strict rules [16]. Participants were brought together in a separate, comfortable area, away from the diabetes consultation and away from parents and other healthcare professionals. Each focus group brought together an average of 3.6 ± 0.3 participants, separating young children and adolescents. Exceptionally, one focus group included only 2 patients due to the fortuitous absence of 3 others that day; in another focus group, 3 patients were added to the 5 planned.

The focus groups were all conducted under the sole egis of the same two persons (SLF and NF), in the vicinity but not inside the Ariane centers. Neither of them is a healthcare professional. They were known by the Ariane doctors for their ability to listen, their organizational skills, their availability, and their experience in clinical research in pediatric diabetology. At the start of each focus group, they explained the objective to the participants: “to find out more about what they think of their diabetes treatment, and their main concerns about it”. They then led discussions around the points spontaneously raised by the children. While avoiding a rigid question-and-answer procedure, they gently guided the children to encourage their oral expression, focusing on how they felt about their treatment. The focus groups were therefore conducted as semisupervised or semistructured interviews. The list of questions used to elicit children's expression (Figure S2) was compiled from the authors' experience of listening to young patients in consultation. Each of the authors brought in topics from their own patients, and the Ariane group chose the list to be used in the focus groups. All children expressed themselves, and most enjoyed taking part. A balance between the talkative and the shy participants was maintained by the animators. The sessions lasted around 2 h and were recorded with a tape recorder. Each child's expression was faithfully transcribed by NF in writing (verbatim) from the recordings.

### 2.4. Analysis of Verbatims

The 3640 verbatims from the focus groups (average 42.8 per participant) were forwarded to people from a variety of backgrounds who were asked to summarize them into a minimal set of 30 points that they felt summed up the main concerns of children and adolescents regarding their treatment. A total of 30 people were co-opted for this analysis: (i) a group of 6 pediatric diabetologists from the Ariane group; (ii) a group of 6 young adults with T1D since childhood; (iii) a group of 5 specialized nurses, whose job it is to visit patients at home; and (iv) two groups of 6 and 7 people from a variety of professional backgrounds having nothing to do with T1D. The 30-point summaries produced by these five groups revealed many similarities. They were compared to extract common points, given the very wide overlap of syntheses by the different groups: 22 points were chosen by all five groups and 2 by four groups of different nature, leading to our 24-item list. A global synthesis of the groups' work was thus a list of 24 points. A patient's BOT score was defined as the number of points (among the 24) by which he or she felt concerned.

### 2.5. Statistical Analysis

Statistical analysis was performed using R. Continuous variables were presented as mean ± SD, and categorical variables were presented as frequencies (%). Differences between groups of continuous variables were analyzed using the nonparametric unpaired two-sample Wilcoxon test. Associations between categorical variables were analyzed using the chi-squared test (*χ*^2^). The intraclass correlation (ICC) is a measure of the agreement between the five groups of observers. An ICC of 0 means that there is no agreement at all, and an ICC of 1 means that there is complete agreement. Generally, ICC values above 0.75 are considered to be good, and values above 0.9 are considered to be excellent.

### 2.6. Data and Resource Availability

The datasets generated and analyzed in the current study are available from the corresponding author upon reasonable request.

## 3. Results

### 3.1. Analysis of Verbatims

**Figure 1:**
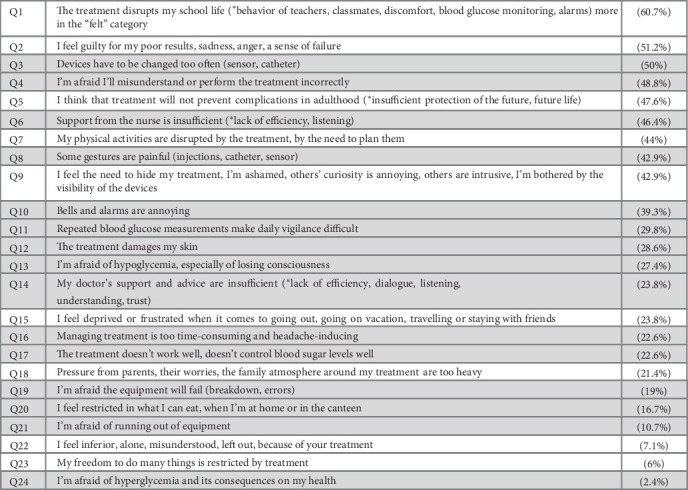
The 24 concerns derived from the analysis of focus group verbatims, presented in descending order of frequency. For each concern, the percentage of patients is noted in brackets. Gray lines are concerns linked to the direct physical consequences of the treatment. White lines are concerns related to psychological aspects in the broad sense.

Two categories of concerns emerged from our analysis (Figure 1): (i) 15 concerns were related to direct physical consequences of treatment, technical or practical aspects of treatment and organization of care, appear in Figure 1; (ii) 9 concerns were related to psychological aspects in the broadest sense, feelings and perception experienced by patients regarding treatment. In this last category (Figure 1), some of the feelings are inevitably intermingled with feelings related to T1D itself (“burden of disease”).

Verbatims coming from a single patient have not been included in the summary analysis because they were too diverse and too individual or anecdotal to be presented.

### 3.2. BOT Score

Children and adolescents expressed a variable number of concerns among the final 24 of the global synthesis. This number was used to calculate a BOT score. The mean BOT score was 7.4 ± 3.3, ranging from 1 to 18. The percentage of children corresponding to each level of BOT score is presented in Table 2. For example, 44/84 (52.4%) patients expressed more than six concerns among the 24, giving a BOT score >6.

**Figure 2:**
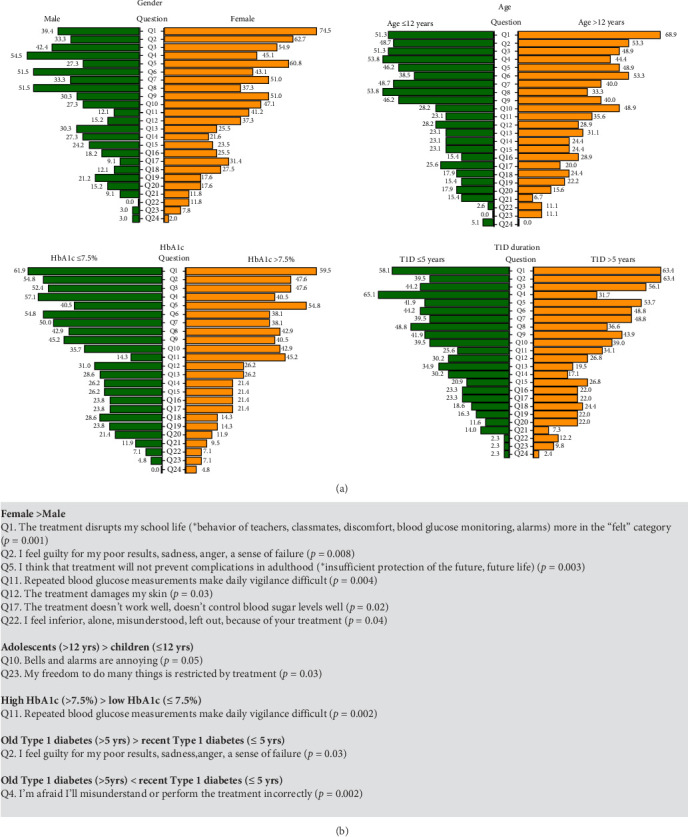
(A) Percent of patients having expressed a given concern. Concerns are presented in increasing order from Q1 to Q24. For example, 74.5% of girls expressed Q1 vs 30.4% of boys. (B) Statistically significant differences (*p*  < 0.05) between categories.

We tested if gender, age, HbA1c, or diabetes duration were associated with each of the 24 concerns (Figure 2), as well as with the global BOT score. Univariate analyses found that the most significant of these factors was gender (*p*=0.02). Indeed, among the 44 patients with a BOT score of >6, girls were 35 (79.5%) and boys only 9 (20.5%) (*p*  < 0.001). The proportions of girls and boys also differed for certain specific concerns, notably psychological feelings (Figure 2). For example, 60.8% of girls unexpectedly expressed Q5 “I think that treatment will not prevent complications in adulthood” versus 27.3% of boys (*p*=0.003). See other gender-dependent concerns in the gray box of Figure 2.

BOT score was not associated with HbA1c (Table 2). However, 45.2% of participants with an HbA1c >7.5% (58.5 mmol/mol) expressed Q11 “Repeated blood glucose measurements make daily vigilance burdensome” versus 14.3% of participants with an HbA1c ≤7.5% (*p*=0.002).

Patients with recent or more ancient T1D had a comparable BOT score (Table 2). However, for some specific concerns, diabetes duration played a significant role (Figure 2). For example, 65.1% of those with a recent T1D diagnosis expressed Q4 “I'm afraid I'll misunderstand or perform the treatment incorrectly” vs. 31.7% of those with more ancient diagnosis (*p*=0.002).

Significant associations are presented in the gray box of Figure 2.

A multivariate linear regression evaluated the relative contributions of age, gender, HbA1c, and T1D to the BOT score (Table S1). It confirmed a strong relationship between BOT score and gender, with a BOT score in boys inferior, on average, by 2.4 compared to girls, for a constant level of age, HbA1c, and diabetes duration (*p*-value = 0.001).

## 4. Discussion

Children and adolescents with T1D are treated with the aim of achieving maximal glycemic efficacy. With this objective in mind, numerous articles and magazines promote intensive multi-injections, pump or closed-loop treatment, recommend that children and adolescents look at their glucose values several times a day, calculate the carbohydrate content of their meals and insulin doses, and plan their physical activities. Fairly little consideration is given in clinical research publications to children's ability to cope psychologically with treatment, a common belief of doctors being that tight glycemic control is associated with satisfaction and psychological comfort. Representative examples can be found in [17–20]. During exchanges with doctors or nurses, it is often the parents who speak for their child, while the latter often remains reserved or even silent about his treatment, except in periods of crisis when nonacceptance of treatment comes to light.

In the literature, BOT in adult patients with chronic illnesses has been the matter of numerous articles (reviewed in [21–25]). In contrast, articles dealing with BOT are scarce in the pediatric literature and deal with cancer [26], asthma [27], hemophilia [28], or growth hormone treatment [29]. Our search on PubMed and Google Scholar with the keywords, “burden of treatment, children, adolescents, and type 1 diabetes” retrieved only a handful of articles [30–33].

Accordingly, in a recent review [34], the authors wrote that they knew of almost no specific publication dedicated to the BOT in children or adolescents with T1D. While the question of BOT in children or adolescents with T1D is often overlooked by clinicians, the literature is more abundant concerning the burdens of parents and caregivers (reviewed in [35–37]).

Contrasting with obedience to doctor-issued directives and voluntary discipline, carelessness is known to reign existentially in childhood and adolescence. Obedience and discipline thus vary widely among patients and are largely dependent on BOT. Adolescence is a vulnerable period for depression, the frequency of which is increasing in today's world, even more in adolescents with T1D [38]. Resulting risks, particularly suicide, are not negligible [39]. Better understanding and reducing the BOT might have a positive impact on the risk of depression.

The current survey is in no way intended to disqualify intensive treatment, a major advance for pediatric T1D. It does, however, aim to take a closer look at how young patients feel about their treatment, in an attempt to minimize the sources of physical and psychological discomfort this treatment creates. Unlike other technologies aiming at intensive treatment, closed-loop systems are often presented as alleviating mental BOT, even though no serious study has convincingly demonstrated this in the long term in a representative population. As a recent review wisely points out, “Recent studies have demonstrated that closed-loop systems offer significant benefits in terms of improved glycemic control. However, these benefits are counterbalanced by important challenges, ranging from variable levels of trust to concerns about physical bulk, technical glitches, and difficulties incorporating closed-loop systems into everyday life”[40].

Our focus group approach combines freedom of expression with rigorous methodology. It complements qualitative approaches based on in-depth personal interviews with psychologists [41, 42].

The main concerns expressed by children and adolescents fall into two categories. On one hand, they highlight concrete, material aspects of treatment and how it fits into daily life and relationships with healthcare professionals. On the other hand, children and adolescents expressed negative psychological feelings about certain aspects of treatment. Our study provides an estimate of the proportion of children expressing each concern. The nature of these concerns will come as no surprise to the majority of pediatric diabetologists, who hear them in their consultations, provided they ask their patients. But the frequency and ranking of concerns are new. Also, the absence of certain aspects that doctors would have expected to be raised, such as physical activity restrictions or projections into the socio–professional or sentimental future, came as something of a surprise.

A major weakness of our study is that it does not quantify the intensity with which participants feel concerns about BOT, since the BOT score that we defined is based solely on the number of concerns expressed by each participant, not on the strength of their feelings. It allowed us, however, to estimate the proportion of children or adolescents who express the most concerns and possibly suffer the greatest BOT. Another weakness is the relatively small size of our cohort, which must temper our vision of frequencies and may question its representativeness. Notably, by selecting patients able and willing to express themselves, our study did not include the silent, introverted patients. In addition, our analysis lacks important parameters, such as the socio–economic and educational background of families, or demands from doctors, or the content of educational programs.

Despite the limited size of our cohort, we attempted to test whether certain patient characteristics conditioned the 24 concerns composing BOT (Figure 2). The best-controlled patients showed no major difference in their BOT concerns when compared to patients with poorer glycemic control (Table 2). One might also have thought that patients would be more anxious in the early years of their treatment, or that older patients would have become weary of treatment, or have had different concerns. But this was not generally the case (Table 2), even if some specific concerns were found to be dependent on diabetes duration (Figure 2). It was unexpected to see how different the proportion of girls and boys was for certain concerns, such as those reflecting psychological feelings (Figure 2). Indeed, the multivariate linear regression analysis showed that the most significant determinant of differences in BOT score appeared to be gender (*p*=0.002, Table S1), girls having much more concerns than boys. We have no explanation for this observation, which does not seem to be due to differences in emotional expression between genders [43]. Also, boys consistently report more depressive symptoms than girls in the general population [44].

In order to assess not only the frequency but also the intensity of these feelings and more extensive association with cited parameters, we are undertaking a large-scale online study of BOT among young patients with T1D, based on the 24 concerns raised by the current study and transformed into a question. In this survey, there will be no exclusion criteria, and we will record the feelings of a large pediatric population in anonymous conditions. This should enable us to identify factors associated with BOT more robustly. To estimate concern intensity, participants will be asked to rank their answers on a Likert scale reflecting the intensity of each concern. They will also be asked to add other major concerns if they have.

## 5. Conclusions

Patient-centered methods such as MDM and shared decision making should join technological advances, patient empowerment, and self-management support [45], BOT should be included in clinical practice [46]. In the current study, the focus group technique proves its feasibility in children and adolescents with T1D. It provides a new tool for assessing BOT, easily applicable to T1D care to serve as a basis for applying minimally disruptive medicine and for the comparative evaluation of therapeutic strategies.

## Figures and Tables

**Table 1:** Main characteristics of the studied 84 patients.

Parameters	Total	Boys	Girls
*N*	84	33	51
Age (year)	12.6 ± 3.7 (5–20)	12.5 ± 3.5 (5–20)	12.7 ± 3.6 (7–20)
HbA1c (%)	7.7 ± 0.4 (6–13.6)	7.6 ± 0.6 (6–9.2)	7.9 ± 1.4 (6–13.6)
HbA1c (mmol/mol)	60.9 ± 4.4 (42–125)	59.8 ± 6.6 (42–77)	62.9 ± 15.3 (42–125)
Type 1 diabetes duration (year)	5.7 ± 3.7 (1–19.5)	5.3 ± 4 (1–17.5)	6.0 ± 3.6 (1.3–19.5)

*Note:* The mean ± SD (range).

**Table 2:** BOT score values according to gender, HbA1c level, or diabetes duration.

Patientsʼ groups	*N*	BOT score	*p* -Value
Total	84	7.4 ± 3.3	—
Gender			
M	33	5.9 ± 3.0	0.0007
F	51	8.3 ± 3.1	
Age (year)			
≤12	39	7.0 ± 3.2	0.39
>12	45	7.6 ± 3.4	
HbA1c (%)			
≤7.5	42	7.7 ± 3.4	0.60
>7.5	42	7.0 ± 3.1	
Diabetes duration (year)			
≤5	43	7.2 ± 3.2	0.72
>5	41	7.5 ± 3.4	

*Note:* The data are mean ± SD. The nonparametric unpaired two-sample Wilcoxon test was applied to compare groups and generate *p*-values.

## Data Availability

The datasets generated and analyzed in the current study are available from the corresponding author upon reasonable request.

## Supporting Information

Supporting Information Table S1. Multivariate linear regression to evaluate relative contributions of age, gender, HbA1c, and T1D duration to the BOT score. The Table S1 below gives the estimate for each parameter, together with the *p*-value of the nullity of the parameter. There is a significant relationship between the BOT score and gender: in boys, the BOT score is lower, on average, by 2.43 compared to girls, for a constant level of age, HbA1c, and diabetes duration (*p*=0.002). The coefficient of determination R^2^ for this model is 0.131, which means that only 13.1% of the variability of the BOT score is explained by gender, age, HbA1c, and T1D duration. Figure S1. Diagram of the Ariane study in continental France and La Réunion island. Figure S2. Focus groups procedure.
