# Supplementary material for: Burden of Treatment in Children and Adolescents With Type 1 Diabetes Evaluated by Focus Groups
Source: Pediatr Diabetes. 2025 Jun 12;2025:8833434. doi: 10.1155/pedi/8833434 (PMC12178731; doi:10.1155/pedi/8833434)
Supplement: Supporting Information — Table S1. Multivariate linear regression to evaluate relative contributions of age, gender, HbA1c, and T1D duration to the BOT score. The Table S1 below gives the estimate for each parameter, together with the p-value of the nullity of the parameter. There is a significant relationship between the BOT score and gender: in boys, the BOT score is lower, on average, by 2.43 compared to girls, for a constant level of age, HbA1c, and diabetes duration (p=0.002). The coefficient of determination R2 for this model is 0.131, which means that only 13.1% of the variability of the BOT score is explained by gender, age, HbA1c, and T1D duration. Figure S1. Diagram of the Ariane study in continental France and La Réunion island. Figure S2. Focus groups procedure. [file 8833434.f1.docx]

**ONLINE-ONLY SUPPLEMENTAL MATERIAL**

**Suppl Table S1.** Multivariate linear regression to evaluate relative contributions of age, gender, HbA1c and type 1 diabetes duration to the BOT score. The table below gives the estimate for each parameter, together with the p-value of the nullity of the parameter. There is a significant relationship between the BOT score and gender: in boys, the BOT score is lower, on average, by 2.43 compared to girls, for a constant level of age, HbA1c and diabetes duration (p =0.002).

The coefficient of determination R^2^ for this model is 0.131, which means that only 13.1% of the variability of the BOT score is explained by gender, age, HbA1c and type 1 diabetes duration.

|  | **Estimate** | **Standard error** | ***P*** |
| --- | --- | --- | --- |
| **(Intercept)** | 9.71 | 3.03 | *0.002* |
| **Age** | -0.02 | 0.14 | *0.91* |
| **Gender** | -2.43 | 0.71 | *0.001* |
| **HbA1c** | -0.16 | 0.36 | *0.66* |
| **Diabetes duration** | 0.01 | 0.12 | *0.96* |

**Suppl Figure S1.** Diagram of the Ariane study in continental France and La Réunion island.

**Suppl Figure S2.** Focus groups procedure.

**
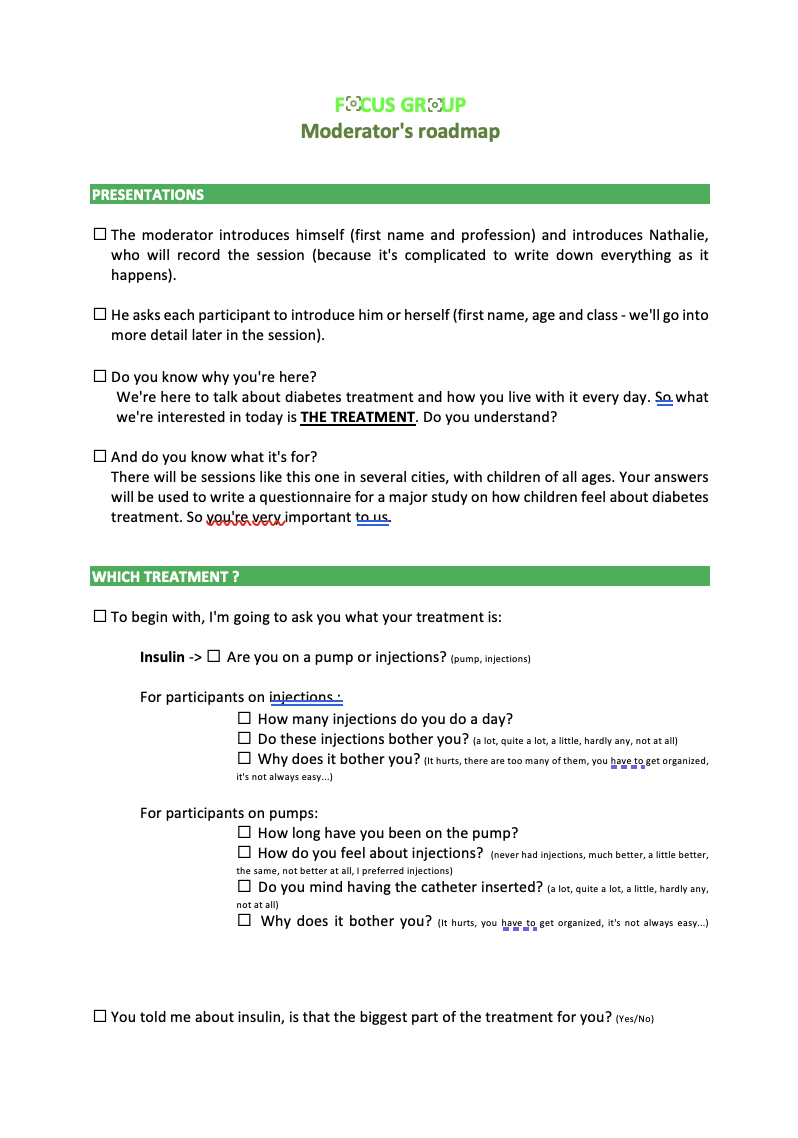
**

**
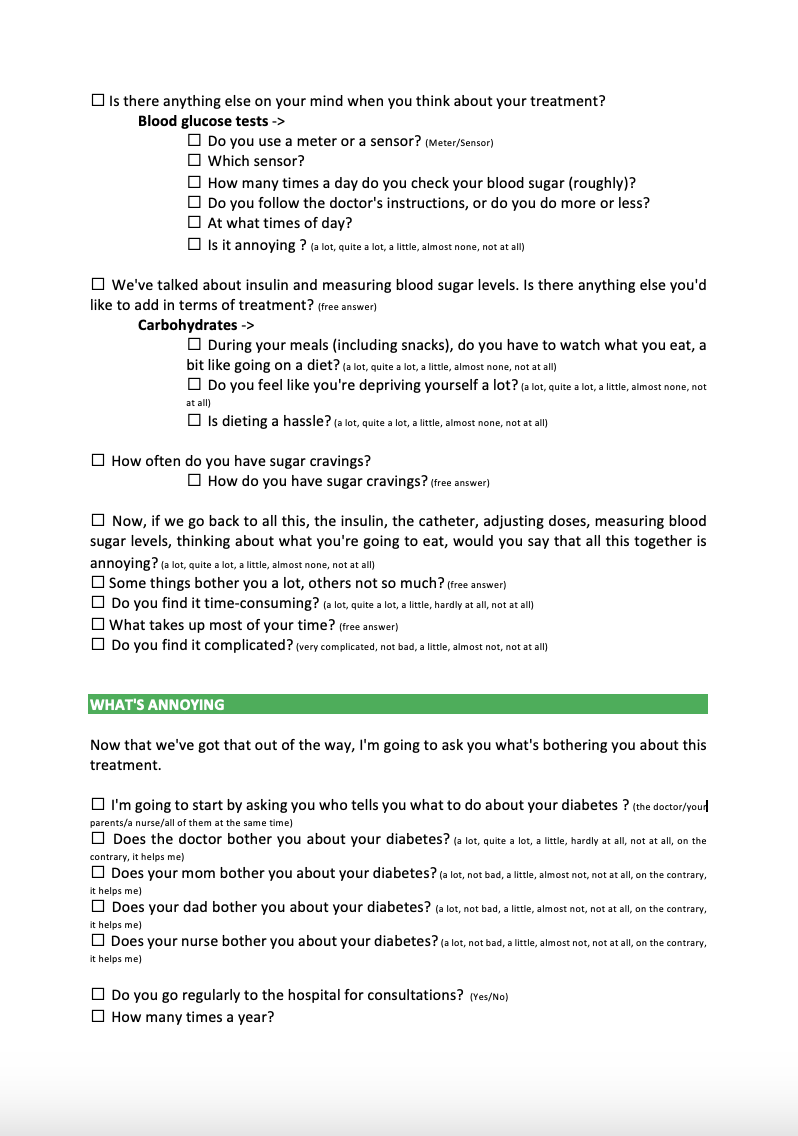
**

**
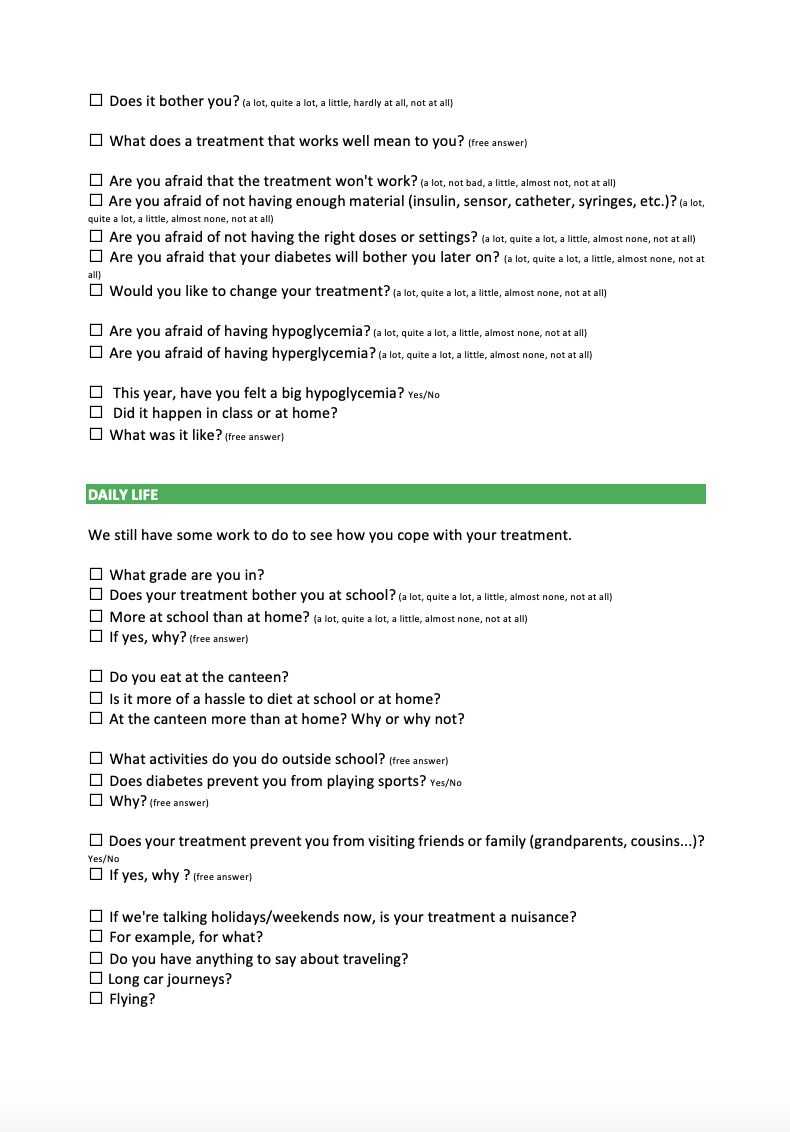
**

**
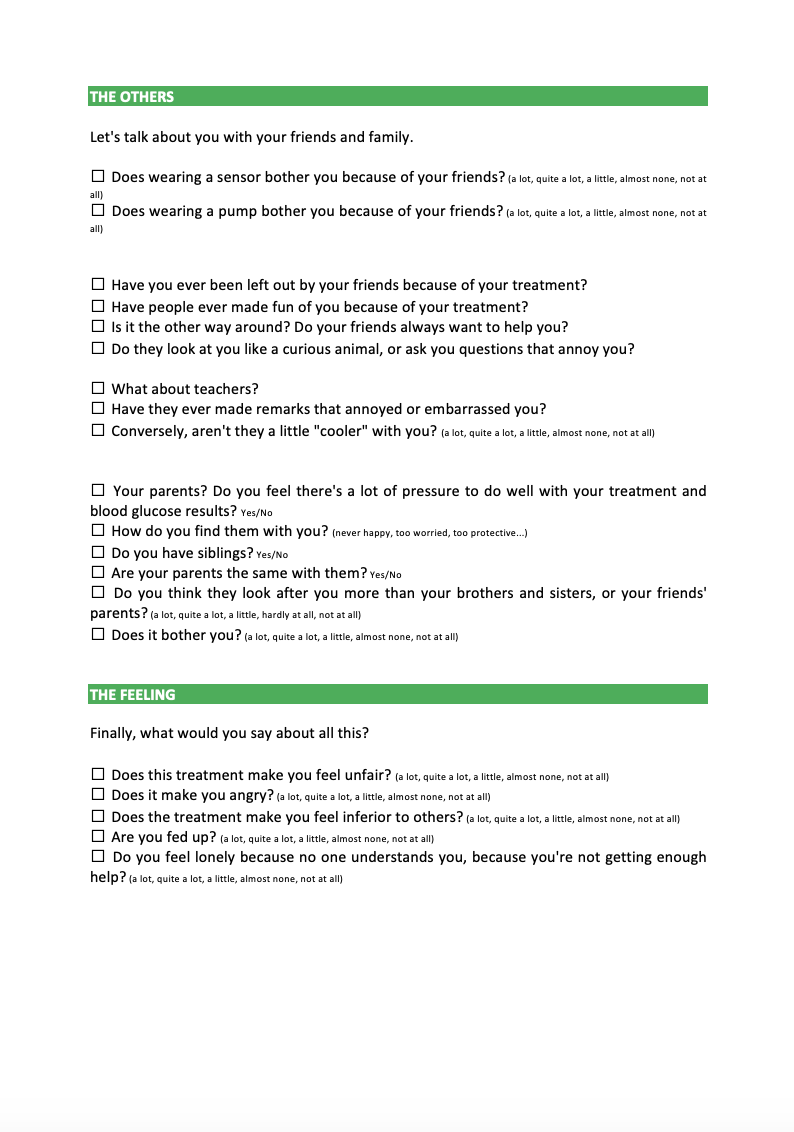
**

**
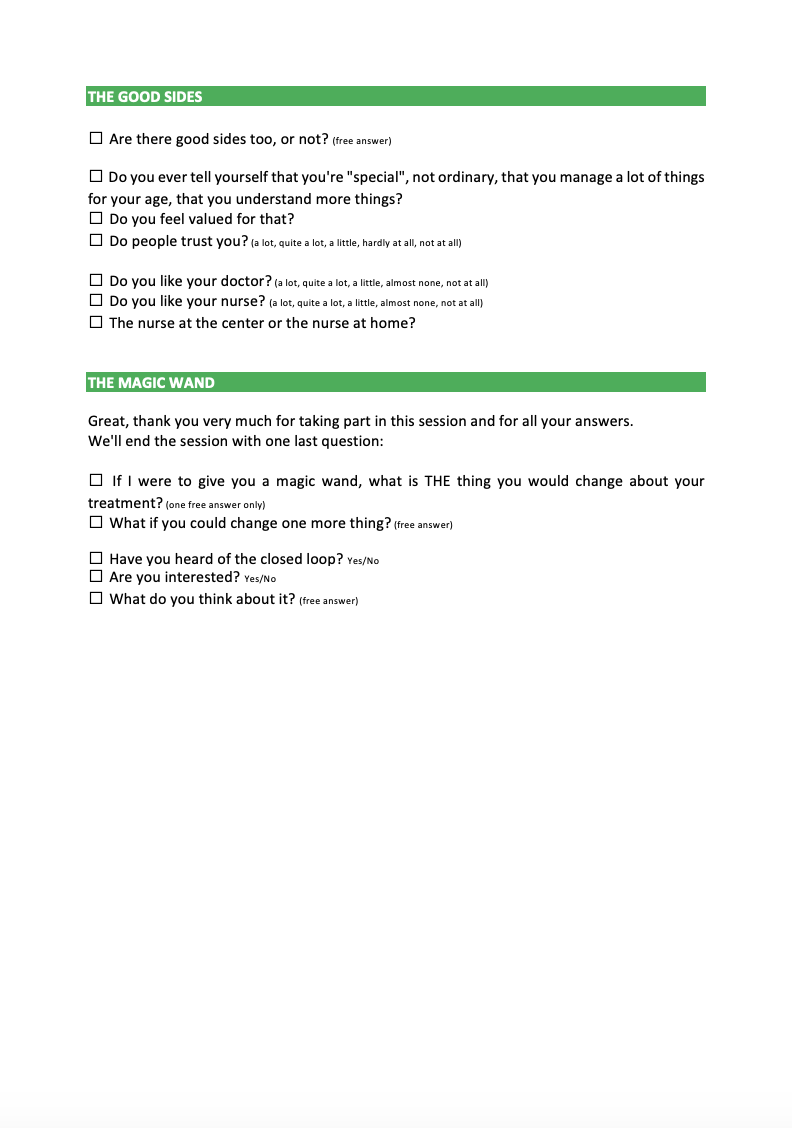
**
